# Media regulation strategies in parents of 4- to 16-year-old children and adolescents: a cross-sectional study

**DOI:** 10.1186/s12889-023-15221-w

**Published:** 2023-02-21

**Authors:** Tanja Poulain, Christof Meigen, Wieland Kiess, Mandy Vogel

**Affiliations:** 1 LIFE Leipzig Research Center for Civilization Diseases, Leipzig University, Philipp-Rosenthal-Strasse 27, 04103 Leipzig, Germany; 2 Department of Women and Child Health, Hospital for Children and Adolescents and Center for Paediatric Research (CPL), Leipzig University, Liebigstrasse 20a, 04103 Leipzig, Germany

**Keywords:** Media regulation, Children, Parents, Socio-economic status, Screen time, Leisure

## Abstract

**Background:**

Since children can only control and limit their own media use to a limited extent, it is often the parents who regulate their children’s media use. However, there is insufficient research on which strategies they use and on how these strategies are related to socio-demographic and behavioral parameters.

**Methods:**

The parental media regulation strategies co-use, active mediation, restrictive mediation, monitoring, and technical mediation were assessed in a sample of 563 four- to 16-year-old children and adolescents from middle to high social strata participating in the German cohort study LIFE Child. We investigated cross-sectional associations with socio-demographic characteristics (age and sex of child, age of parent, and socio-economic status (SES)) and other behavioral parameters of children (media use, ownership of media devices, engagement in extracurricular activities) and their parents (media use).

**Results:**

All media regulation strategies were applied frequently, with restrictive mediation occurring most frequently. Overall, parents of younger children and of boys mediated media use more frequently, while we observed no differences depending on SES. Regarding child behavior, the ownership of a smartphone and a tablet/personal computer/laptop was associated with more frequent technical restriction, while screen time and engagement in extracurricular activities was not associated with parental media regulation. In contrast, parental screen time was related to more frequent co-use and less frequent use of restrictive and technical mediation.

**Conclusion:**

Parental regulation of child media use is influenced by parental attitudes and a perceived need for mediation (e.g., in younger children or children owning internet-enabled devices) rather than child behavior.

**Supplementary Information:**

The online version contains supplementary material available at 10.1186/s12889-023-15221-w.

## Background

The use of electronic media in children and adolescents has increased dramatically in the last years [1]. This trend is disturbing, given that some media activities, e.g., playing computer games, have a potential for addiction [2]. An excessive use of electronic media in childhood and adolescence has also been shown to be associated with mental health problems [3, 4], sleep disturbances [5–7], and behavioral difficulties [8, 9].

One reason why the use of electronic media bears the risk of addiction in children is their limited ability to control their media use and their reduced awareness of potential negative consequences of excessive use [10]. Parents often view their children’s media use more critically than the children themselves and try to regulate their children’s media use. Parental media regulation has been defined as strategies parents use to control, supervise, or interpret media content [11]. When television was still the main media activity that children pursued, the following media regulation strategies were distinguished: *co-use* (e.g., watching films and series together), *active (or instructive) mediation* (e.g., speaking about content of films and series), and *restrictive mediation* (e.g., limiting the time a child spends in front of the TV) [12, 13]. While these media regulation strategies can be adapted to the use of modern media such as mobile phones and computers, they do not cover all possible mediation strategies. Therefore, two other strategies have been described and investigated in the recent past: *monitoring* (e.g., controlling which internet sides a child visits) and *technical mediation* (e.g., stopping internet access at a specific time) [14]. However, several studies still focus on the three more traditional media regulation strategies. Therefore, there is little evidence to date on associations between monitoring or technical restriction and sociodemographic or behavioral parameters.

In European families, active mediation and restrictive mediation represent the media regulation strategies applied most frequently [15]. Regarding relationships between parental media regulation and socio-demographic parameters, previous findings indicate that media regulation, especially restrictive mediation, is applied more frequently in younger than in older children [15–17]. While previous studies revealed no considerable sex differences [15, 18], media regulation has partly been shown to differ depending on socio-economic status (SES). Active [15] and technical mediation [18] seem to be more common in families with a higher SES, while restrictive mediation has been shown to be more frequent in families with a middle or lower SES [17].

With respect to possible links between media regulation and the media behavior of children and parents, a previous study in preschool-aged children showed significant associations between restrictive mediation and negative attitudes towards media and shorter screen times of parents [18]. In contrast, the same study revealed no significant associations between children’s media use and parental media regulation [18]. In older children and adolescents, active mediation was associated with lower amounts of problematic internet use in several studies, while co-use and monitoring showed no significant associations with internet use [19]. Regarding restrictive mediation, the findings of previous studies were mixed [19]. Overall, these findings suggest that media regulation is a part of parent’s educational style that shows only a limited connection to the actual media use of children.

Whether or not parental media regulation is associated with other leisure activities of children is not explored. According to the displacement hypothesis, high electronic media use may displace other activities such as doing homework or social and physical activities [20]. Therefore, one might also suggest that the participation in extracurricular activities, e.g., participating in sports clubs or music groups, structure children’s everyday life and, therefore, limit or regulate children’s media use without parental involvement becoming necessary. In that case, participating in extracurricular activities might be linked not only to children’s media use but also to parental media regulation strategies. However, to the best of our knowledge, no previous study investigated the association between the engagement in extracurricular activities and media regulation of parents.

The aim of the present study was to investigate the frequency of the five media regulation strategies (co-use, active mediation, restrictive mediation, monitoring, and technical mediation) in a sample of German families. Furthermore, we explored associations with socio-demographic factors (child age and sex, age of parents, and SES) as well as behavioral factors of children (media use and ownership of media devices, engagement in extracurricular activities) and parents (media use). Based on previous study findings, we expected media regulation to be more frequent in parents of younger children, but less frequent in parents showing a high media use. Based on our own assumptions, we also expected less frequent media regulation in parents whose children engage in extracurricular activities.

## Methods

### Participants

The present study was conducted as part of the LIFE Child study, an ongoing cohort study that has been conducted at the Leipzig University in eastern Germany since 2011. LIFE Child aims to monitor the development of healthy children from the prenatal period to young adulthood, with a specific focus on obesity and mental health [21]. Participants are mainly recruited by word of mouth and by advertisement at different health institutions. All children not suffering from any syndromal diseases are invited to participate. By the end of 2021, more than 5000 children and adolescents have participated in the LIFE Child study, resulting in more than 20.000 study visits.

For the present project, data were collected in 2021, when the new media use questionnaires were added to the study program. All children whose parents had completed the questionnaire on parental strategies to regulate children’s media use were eligible for analysis (n = 712 four- to 16-year-old children). In the case that children did not use any screen-based media (n = 64) and in the case that parents stated media regulation to be “not applicable” (e.g., if the child uses media in such a way that the corresponding strategy cannot be implemented, n = 85), children were excluded from analysis. The final sample comprised 563 children (298 male, 265 female) aged 4 to 16 years (mean age = 11.1, sd = 3.2). Due to assessment-specific missings, analyses regarding associations between media regulation strategies and engagement in extracurricular activities were performed in a slightly smaller sample (n = 526). For analyses on associations between media regulation and screen time of parents, we only included one child per family (the youngest), resulting in a reduced sample (n = 366). In this sample, the mean age of children was 10.51 (sd = 3.3).

The LIFE Child study protocol was designed in accordance with the Declaration of Helsinki and was approved by the Ethics Committee of the Medical Faculty of Leipzig University (Reg. No. 264/10-ek). All parents provided informed written consent before the participation of their children. Children themselves gave their verbal consent (up to the age of 11 years) or their written consent (from the age of 12 years).

### Measures

#### Media regulation

Parental media regulation was assessed based on five questions, each capturing one media regulation strategy (co-use, active mediation, restriction, monitoring, and technical mediation). The questions are presented in Additional file 1. Responses were given on a five-point Likert scale (ordinal scale), with response options ranging from never to always (see Additional file 1).

#### Media use of children and parents

For the assessment of parental screen time, parents were asked to estimate the minutes they spend using screen-based media on a weekday and on a weekend day (excluding use for work). They could choose between 25 half-hour intervals (e.g., “0 minutes”, “0.5 hours” […] “11.5 hours”, “12 hours”). For further analyses, the responses were transformed to minutes per day (e.g., “0”, “30” […] “690”, “720”), and screen times on weekdays and weekends were combined to the (metric-scaled) variable screen time per day ((minutes on weekday*5 + minutes on weekend*2)/7).

The same procedure was applied for the assessment of children’s screen time. For children up to the age of 10.5 years, screen time was estimated by parents (n = 332). In the course of the LIFE Child study, it has been shown that children younger than 10.5 years are not able to grasp questionnaire texts and to answer them quickly and correctly. Older children self-rated their screen time (n = 380). Ownership of a smartphone, a tablet/personal computer (pc)/laptop or a television in the bedroom was determined by asking children (10.5 years or older) or their parents (for younger children) whether or not they already owned the respective device. The exact questions and response options are presented in Additional file 1.

#### Participation in extracurricular activities

Engagement in extracurricular activities was assessed using two questionnaires completed by children themselves (10.5 years or older) or their parents (for younger children). In a physical activity questionnaire, they were asked to indicate whether or not children participate in organized sports. In a leisure activity questionnaire, they indicated whether or not they were engaged in a music group, e.g., orchestra or choir, in a theatre or dance group, or in another extracurricular activity. They were also asked to indicate whether or not they play an instrument. For further analysis, all children engaging in at least one of these activities were categorized as engaging in an extracurricular activity. The exact questions and response options are presented in Additional file 1.

#### Socio-economic status (SES)

A SES composite score combining information on parents’ education, occupation, and income was used to assess the socio-economic status (SES) of the participating families [22]. Values of the score range from 3 to 21, with higher values indicating higher SES. The score can be used to categorize a family’s SES as either low, middle, or high. In a representative sample, the distribution of these groups would be expected to be 20-60%-20% [22].

### Statistical analysis

This is a cross-sectional study. All statistical analyses were performed using R [23]. Metric variables were described by means (m) and standard deviations (sd), while ordinal or binary variables were described by absolute and relative frequencies. Correlations between media regulation strategies were calculated using Spearman rank-order correlations.

To assess associations between parental media regulation and the socio-demographic parameters age of child, age of mother, sex of child, and SES of the family (as continuous measure ranging from 3 to 21), we applied ordinal mixed effects models. The media regulation strategy was included as ordered ordinal dependent variable, and the socio-demographic parameters were included simultaneously as independent variables. These analyses were assessed in the large sample of 563 children. To control for family relationships within the sample (siblings), the family id was included as random effect.

In order to investigate associations between parental media regulation (dependent variable) and screen time of children (n = 563), ownership of smartphone, tablet/pc/laptop, or television in the bedroom (n = 563), and engagement in extracurricular activities (n = 526), we also applied ordinal mixed effect models controlling for family relationships within the sample. All associations were adjusted for socio-demographic parameters that had shown a significant association with at least one media regulation strategy (namely age of child, age of parent, and sex of child).

Associations between parental media regulation (dependent variable) and parental screen time was performed in the smaller sample of children (excluding siblings, n = 366). Here, we applied ordinal regression analyses. Again, associations were adjusted for age of child, age of parent, and sex of child.

## Results

The final sample comprised data of 563 four- to 16-year-old children (53% boys, mean age = 11.1 years) and their parents (328 mothers, 38 fathers, mean age = 43.0, range 29–60). The SES of the participating families was rather high (47% high, 52% middle), with an underrepresentation of families from low social strata (1%).

Further characteristics of the study sample are summarized in Table 1. On average, children as well as parents spent 2.5 h per day using screen-based media. While most children owned a tablet/pc/laptop (72%) and a smartphone (62%), televisions in bedroom were less frequent (23%). The majority of children (79%) engaged in at least one extracurricular activity.

**Table 1:** Characteristics of the study sample

Variable	N		
Sex	563	n (%)	298 (53%) male, 265 (47%) female
Age	563	mean (sd)	11.1 (3.24)
SES	563	n (%)	263 high (47%), 295 (52%) middle, 5 (1%) low
Sex parents	366	n (%)	328 (90%) female, 38 (10%) male
Age parents	366	mean (sd)	43.0 (5.83)
Screen time children	563	mean (sd)	2.66 (1.91)
Screen time parents	366	mean (sd)	2.54 (1.81)
Ownership smartphone	563	n (%)	214 (38%) no, 349 (62%) yes
Ownership tablet/pc/laptop	563	n (%)	156 (28%) no, 407 (72%) yes
Ownership TV	563	n (%)	433 (77%) no, 130 (23%) yes
Extracurricular activities	526	n (%)	116 (21%) no, 410 (79%) yes

### Parental media regulation strategies

**Fig. 1:**
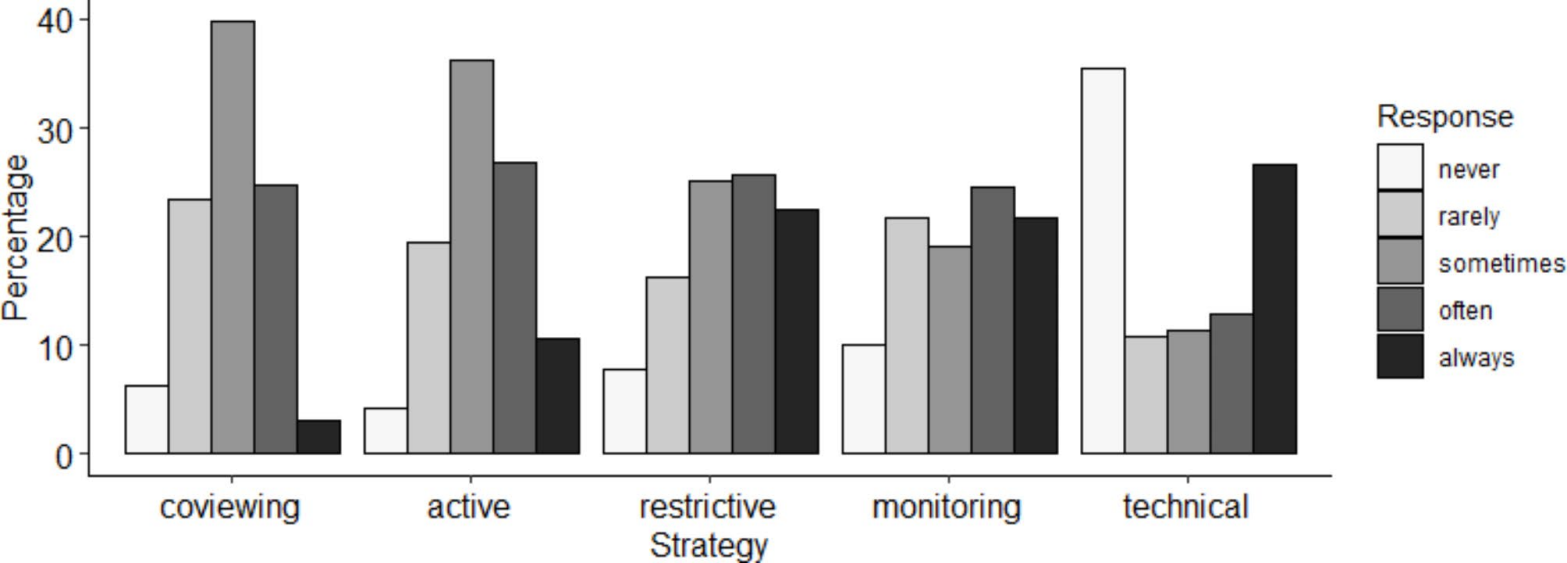
Frequency of different media regulation strategies

The frequencies of the different media regulation strategies are presented in Fig. 1. With the exception of technical mediation, which was most frequently applied either never or always, the media regulation strategies were reported to be applied at least “rarely”, with the response categories “sometimes” and “often” being chosen most frequently. The media regulation strategies restriction and monitoring were reported most frequently. Overall, only four parents (1%) stated to apply none of the five media regulation strategies.

The correlations between the different media regulation strategies are displayed in Table 2. High correlations (> 0.50 [24]) were observed between the strategies of co-use and active mediation and between the strategies of restriction and monitoring. Technical mediation was the only strategy that showed only weak correlations (< 0.30 [24]) with all other media regulation strategies.

As shown by the regression analyses (see Table 3), all media regulation strategies but technical mediation were applied significantly more frequently in younger than in older children. Technical mediation, in contrast, was more frequent in older children. Restrictive mediation, monitoring, and technical mediation were applied more frequently in boys than girls. Regarding the age of parents, younger age was significantly associated with a more frequent use of restrictive mediation and technical mediation. Family SES, in contrast, was not significantly associated with the frequency of any media regulation strategy.

**Table 2:** Spearman rank-order correlations between the frequencies of different media regulation strategies

	Active	Restrictive	Monitoring	technical
Co-use	0.51***	0.20***	0.30***	0.04
Active		0.32***	0.38***	0.14***
Restrictive			0.55***	0.25***
Monitoring				0.17***

***p < .001

**Table 3:** Associations between media regulation strategies and age of child, age of mother, gender, and SES (n = 563)

		Media regulation strategy				
		Co-use	Active mediation	Restrictive mediation	Monitoring	Technical mediation
Age child	OR	0.85	0.87	0.83	0.72	1.08
	95% CI	0.78–0.92	0.80–0.95	0.77–0.89	0.67–0.78	1.00–1.11
	P	< 0.001	< 0.01	< 0.001	< 0.001	< 0.05
Age parent	OR	0.96	0.98	0.94	0.97	0.94
	95% CI	0.91–1.01	0.93–1.03	0.90–0.98	0.93–1.01	0.89–0.98
	P	0.099	0.534	< 0.01	0.166	< 0.01
Gender (female)	OR	0.89	0.80	0.47	0.67	0.56
	95% CI	0.58–1.36	0.51–1.24	0.32–0.68	0.47–0.95	0.36–0.86
	P	0.583	0.316	< 0.001	< 0.05	< 0.01
SES	OR	0.98	0.99	1.00	0.98	1.05
	95% CI	0.90–1.07	0.91–1.09	0.93–1.08	0.91–1.05	0.96–1.13
	P	0.671	0.873	0.913	0.571	0.276

All independent variables were entered simultaneously in the model. To control for family relationships within the sample (siblings), the family id was included as random effect.

### Associations between media regulation and media use of children and parents and engagement in other extracurricular activities

**Fig. 2:**
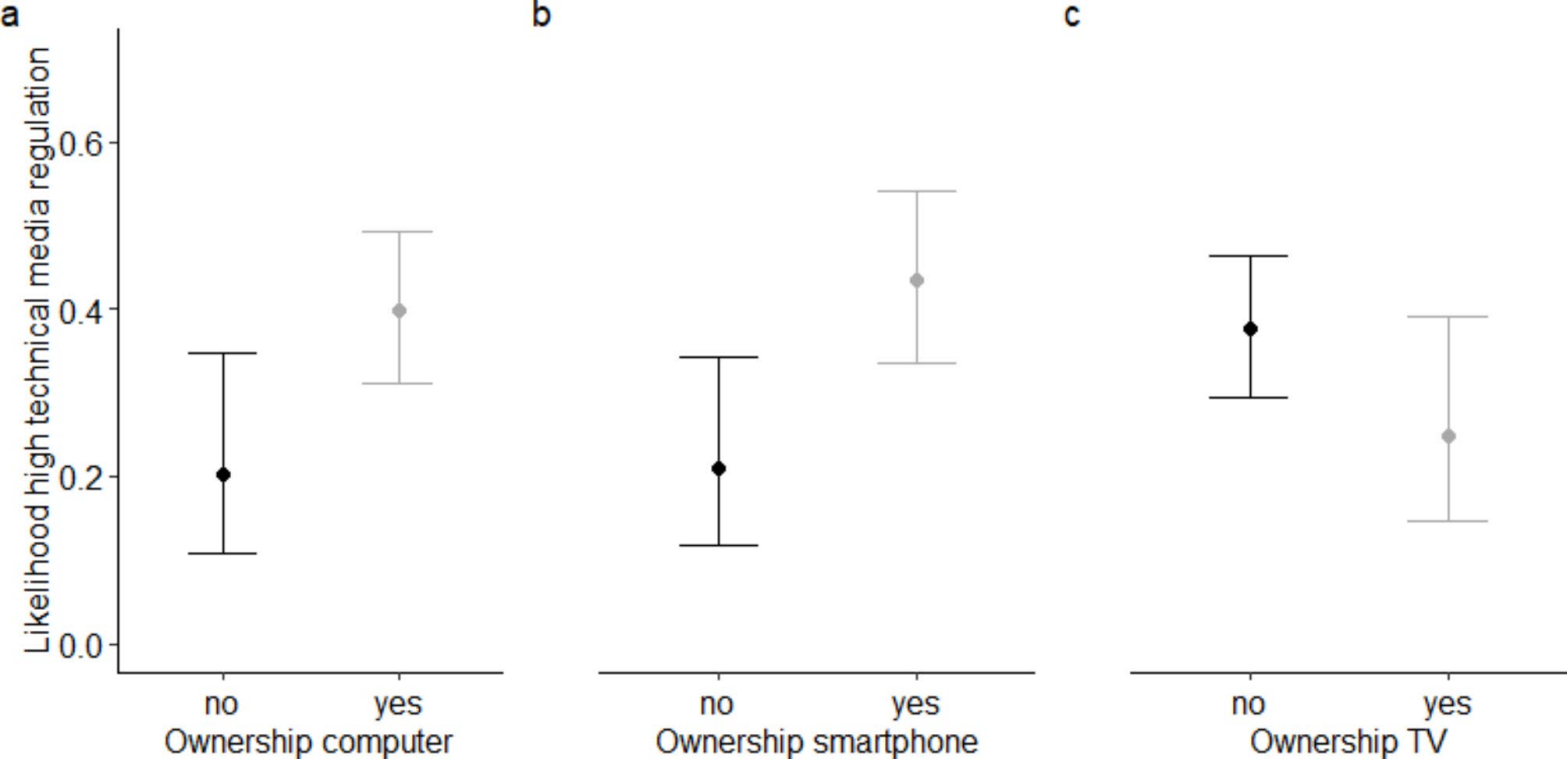
Effect plots illustrating the estimated difference (+ 95% CI) in the likelihood of high technical media regulation (response categories “often” and “always”) depending on the ownership of media devices. While technical mediation was significantly higher if children owned a tablet/pc/laptopr (a) or smartphone (b), it was not significantly associated with the ownership of a TV (c)

While parental media regulation was not significantly associated with children’s engagement in extracurricular activities or their screen time (see Table 4), technical mediation was significantly more frequent if children owned a smartphone (OR = 2.21 (1.16–4.24), p < .05) or a tablet/pc/laptop (OR = 2.40 (1.31–4.42), p < .01), see also Fig. 2. Having a TV in the bedroom, in contrast, was associated with none of the media regulation strategies (see Fig. 2). Regarding the media use of parents, higher screen time was significantly associated with a more frequent co-use of media (OR = 1.14 (1.03–1.27), p < .01) but a less frequent use of restrictive mediation (OR = 0.85 (0.77–0.94), p < .01) and technical mediation (OR = 0.88 (0.79 − 0.98), p < .05). All associations are also presented in Table 4.

**Table 4:** Associations between media regulation strategies and screen time of children and parents, ownership of media devices, and engagement in extracurricular activities

		Media regulation strategy				
		Co-use	Active mediation	Restrictive mediation	Monitoring	Technical mediation
Screen time child (n = 563)	OR	0.96	0.95	0.92	0.94	0.94
	95% CI	0.83–1.10	0.82–1.09	0.82–1.04	0.84–1.05	0.82–1.08
	P	0.542	0.498	0.180	0.288	0.417
Ownership smartphone (n = 563)	OR	0.75	0.81	0.94	0.92	2.21
	95% CI	0.40–1.39	0.52–1.26	0.54–1.63	0.55–1.55	1.16–4.24
	P	0.354	0.352	0.834	0.764	< 0.05
Ownership table/pc/laptop (n = 563)	OR	1.02	1.07	0.83	1.10	2.40
	95% CI	0.68–1.54	0.57–1.99	0.49–1.40	0.68–1.79	1.31–4.42
	P	0.927	0.833	0.485	0.698	< 0.01
Ownership TV	OR	0.59	0.78	0.72	1.03	0.64
(n = 563)	95% CI	0.33–1.06	0.43–1.40	0.47–1.11	0.66–1.61	0.36–1.11
	P	0.080	0.404	0.138	0.897	0.113
Extracurricular activities (n = 526)	OR	0.75	0.99	0.97	1.00	1.12
	95% CI	0.35–1.60	0.54–1.82	0.58–1.63	0.60–1.65	0.63–2.02
	P	0.451	0.966	0.915	0.987	0.701
Screen time parents (n = 366)	OR	1.14	1.01	0.85	0.96	0.88
	95% CI	1.03–1.27	0.92–1.12	0.77–0.94	0.87–1.06	0.79–0.98
	P	< 0.01	0.796	< 0.01	0.451	< 0.05

All associations were adjusted for age of child, age of parent, and sex of child. In the analyses regarding screen time of children, media device ownership, and engagement in extracurricular activities, we controlled for family relationships within the sample (siblings) by including the family id as random effect. The analyses regarding screen time of parents was only conducted in a sample excluding (older) siblings.

## Discussion

### Parental media regulation

The present study investigated parental media regulation strategies in a sample of four- to 16-year-old children growing up in Germany. Overall, nearly all parents applied some media regulation strategies at home, with the restrictive strategies of restrictive mediation and monitoring being reported most frequently. This finding is in line with previous studies showing that, compared to parents from other European countries, parents in Germany feel a high need to “protect” their children from the potential risks of excessive media use and, therefore, often apply restrictive regulation strategies [16, 17]. However, the more active media regulation strategies of active mediation and co-use were also applied relatively frequently. The correlation analyses revealed that the restrictive media regulation strategies (restrictive mediation and monitoring) are often applied in parallel. Similarly, the active regulation strategies (active mediation and co-use) were highly correlated, indicating that they are also often applied in parallel or even simultaneously, e.g., when parents use the shared media time to provide their children with media-relevant information (interactive coviewing [25]). Technical mediation, in contrast, is applied independently of other regulation strategies. This strategy might not primarily reflect parents’ tendency to apply a restrictive or active mediation style but rather their attitude towards or their ability to implement this technical way of mediation.

Our analyses revealed that active and restrictive media regulation strategies were applied more frequently in younger children than in older children. This finding is in line with previous studies [15–17] and might reflect the increasing (medial) independence of older children as well as the increasing powerlessness of parents regarding the regulation of their children’s media use. While younger children are less competent media users and acknowledge or even ask for their parents’ help, older children and adolescents are often more familiar with modern media than their parents and do not accept parental control over their media use. In contrast to the other media regulation strategies, technical mediation was applied more frequently in older as compared to younger children. This finding contradicts observations made in a large European study, which, however, was based on an older sample (9–16 years) [15]. Our finding might suggest that in older as compared to preschool-aged children, limiting access to the internet (the main technical restriction) might represent the most appropriate or even the only possible way to limit media use.

Not only the age of children but also the age of parents was associated with parental media regulation. Restrictive mediation as well as technical mediation were more frequent among younger parents than among older parents. Due to a greater exposure to modern media, younger parents might be more aware of problems that might arise from media use and, consequently, of the need to regulate media use. They might also be more familiar with appropriate strategies, e.g., technical possibilities to limit media use.

While some previous studies did not find any gender differences in parental media regulation [15, 18], our analyses revealed that parents of boys applied media regulation strategies (especially restrictive strategies and technical mediation) more frequently than parents of girls. Boys might be less able to regulate their media use independently. The difference between boys and girls might also be driven by differences in media use. Boys are usually more likely to play video games than girls [26]. As video games have a potential for addiction [2], parents might see a higher need to limit this activity.

Interestingly, our analyses revealed no significant differences in parental media regulation depending on SES. This contradicts previous studies [15, 17, 18]. This observation might be explained by the fact that families of lower social strata were underrepresented in the present study.

### Associations between parental media regulation and media use of children and parents and children’s participation in organized sports and extracurricular activities

In the present study, none of the parental media regulation strategies was associated with children’s screen time. This is in line with a review according to which most parental mediation strategies (with some exceptions for active and restrictive mediation) were not associated with children’s internet usage [19]. The finding could be explained by the fact that parental media regulation might be reactive and proactive. In families in which media regulation represents a reaction to (high) media use, one would expect a positive association between media regulation and screen time. In families that regulate media proactively, one would expect a negative association. Therefore, it is not surprising that, across all families, there is no significant association. A further explanation for the finding is that we did not assess how strong restrictions actually are (some parents might set the time limit at 2 h a day, others at 2 h a week) or whether children actually adhere to parental restrictions. In contrast to screen time, the ownership of media devices was associated with parental media regulation. While some previous studies found a negative association between media devices (especially a TV) in the bedroom and parental media regulation [18, 27], our analyses revealed more frequent technical mediation if children owned (mobile) internet-enabled media devices (smartphone, tablet/pc/laptop). This finding indicates that parents are aware of the need to regulate the use of mobile media and social media apps, which are generally enabled by smartphones and tablets/laptops rather than a TV. Technical mediation (e.g., limiting internet access) might be the most appropriate form to limit these media activities.

Interestingly, while screen time of children was not associated with parental media regulation, parental screen time was. While co-use was more frequent in parents reporting a higher screen time, technical mediation and restrictive mediation occurred less frequently. The latter association was also found in a previous study in Dutch preschool children [18]. Parents who use electronic media more frequently might have a more positive attitude towards media use in children [28] and, therefore, might tend to not restrict the media use of their children. The association between a rather positive attitude towards media and media regulation strategies has been shown in several studies [18, 27, 29].

We expected that parents whose children engage in extracurricular activities might feel a reduced need to regulate their children’s media use, as extracurricular activities structure their children’s day and, therefore, represent a “natural” media use regulator. Contrary to this expectation, children’s engagement in an extracurricular activity was not associated with the frequency of parental media regulation strategies. Taken together with the finding of no associations between media regulation and children’s screen time, the findings suggest that parental media regulation is not influenced by the leisure behavior of children. Whether or not and how parents regulate their children’s media use might be more dependent on parental attitudes (e.g., towards media use in general) or – as the observed associations suggest – the perceived necessity to regulate children’s media use (e.g., because children are too young or might engage in media activities that have the potential for addiction).

### Strengths and limitations

The investigation of five different media regulation strategies and their association with several demographic and behavioral characteristics of children and parents in a large sample covering a wide age range represents a strength of this study. The underrepresentation of families from low social strata, however, limits the generalizability of the study findings. In particular, the relationship between SES and the use of media regulation strategies might be different (stronger) in a representative sample. A further limitation is the reliance on questionnaire data, which might be subject to different biases (e.g., social desirability). Regarding the media device ownership, grouping tablet, laptop and pc together neglects the potential differences among these devices (e.g., in terms of mobility and types of media activities).

## Conclusion

Parents are aware of the need to regulate their children’s media use and apply restrictive as well as active media regulation strategies. The present findings suggest that parental media regulation is more common when parents feel a stronger need and ability to regulate their children’s media use, i.e., when children are younger or own (mobile) internet-enabled media devices. They, furthermore, suggest that parental media regulation is influenced by the media use of parents rather than the leisure behavior (including the media use) of their children. Parents should be empowered to regulate their children’s media use, with more emphasis on active mediation. As it remains unclear which forms of parental media regulation are most effective in preventing problematic media use and how exactly parents should implement these forms in everyday life, more longitudinal studies and more efforts to transfer the findings into practice (e.g., through prevention or intervention programs) are needed.

## Electronic supplementary material

Below is the link to the electronic supplementary material.

Supplementary Material 1

## Data Availability

The datasets generated and/or analyzed during the current study are not publicly available due to ethical restrictions. The LIFE Child study is a study collecting potentially sensitive information. Publishing data sets is not covered by the informed consent provided by the study participants. Furthermore, the data protection concept of LIFE requests that all (external as well as internal) researchers interested in accessing data sign a project agreement. Researchers that are interested in accessing and analyzing data collected in the LIFE Child study may contact the data use and access committee (forschungsdaten@medizin.uni-leipzig.de).

## List of abbreviations

SES: Socio-economic status
PC: personal computer
