# Supplementary material for: Media regulation strategies in parents of 4- to 16-year-old children and adolescents: a cross-sectional study
Source: BMC Public Health. 2023 Feb 21;23:371. doi: 10.1186/s12889-023-15221-w (PMC9942333; doi:10.1186/s12889-023-15221-w)
Supplement: Supplementary file 1 — Supplementary Material 1 [file 12889_2023_15221_MOESM1_ESM.pdf]

Additional file 1

Questions applied in the present study

Manuscript: Media regulation strategies in parents of 4- to 16-year-old children and adolescents: A cross-sectional study

Authors: Tanja Poulain, Christof Meigen, Wieland Kiess, Mandy Vogel

Journal: BMC Public Health

Original questions (German)

Question	Response option
Media regulation	
Wie oft verwenden Sie folgende Strategien, um die Mediennutzung (TV, Smartphone, PC, Tablet, u.ä.) Ihres Kindes zu unterstützen bzw. zu reduzieren?<br>- Gemeinsame Nutzung, z.B. gemeinsam mit dem Kind im Internet recherchieren, zusammen Computerspiele spielen oder gemeinsam Filme/Serien schauen<br>- Aktives Erklären und Unterstützen, z.B. Filminhalte erklären, Erklären, worauf man im Internet achten muss (Sicherheit, Datenschutz etc.), Helfen bei aufgetretenen Problemen/Unsicherheiten<br>- Verbieten/Beschränken (nicht technisch), z.B. Zeit der Nutzung beschränken, bestimmte Inhalte/Aktivitäten/Netzwerke verbieten<br>- Kontrollieren, z.B. Inhalte der Mediennutzung (Filme, Internetseiten) kontrollieren, Prüfen, welche Informationen das Kind in sozialen Netzwerken teilt<br>- Technisch Einschränken, z.B. automatisches Abstellen des W-LANs, sonstige Timer, die die Nutzungsdauer beschränken	nie<br>selten<br>manchmal<br>oft<br>immer
Screen time child	
Wie lange verbringt Ihr Kind pro Tag in seiner Freizeit (Schulaufgaben zählen nicht mit) an einem Wochentag oder an einem Tag am Wochenende insgesamt vor/mit Bildschirm-Medien (TV, Smartphone, PC, Tablet, u.ä.)?<br>- Dauer - Wochentag<br>- Dauer - Tag am Wochenende	Gar nicht,<br>0.5 Stunden<br>1 Stunde<br>1.5 Stunden<br>[…]<br>12 Stunden<br>> 12 Stunden
Screen time mother	
Wie lange verbringen Sie pro Tag in Ihrer Freizeit (Arbeit zählt nicht mit) an einem Wochentag oder an einem Tag am Wochenende insgesamt vor/mit Bildschirm-Medien (TV, Smartphone, PC, Tablet, u.ä.)?<br>- Dauer - Wochentag<br>- Dauer - Tag am Wochenende	Gar nicht,<br>0.5 Stunden<br>1 Stunde<br>1.5 Stunden<br>[…]<br>12 Stunden<br>> 12 Stunden

Device ownership child	
Welche der folgenden Dinge besitzt Ihr Kind persönlich (im eigenen Zimmer)?<br>- Smartphone<br>- Laptop, Tablet o.ä.<br>- PC<br>- Fernseher, Smart-TV o.ä.	ja<br>nein
Extracurricular activities child	
Welche Arten sportlicher Betätigung hat Ihr Kind im vergangenen halben Jahr ausgeübt?<br>- Sport organisiert im Verein	ja<br>nein
Welchen der folgenden Hobbys geht Ihr Kind nach?<br>- Chor, Orchester oder andere Musikgruppe<br>- Theater- oder Tanzgruppe<br>- Instrument spielen<br>- Sonstige AG (außer Sportverein)	ja<br>nein

English translation

**Question**	**Response option**
Media regulation	
How often do you use the following strategies to support or reduce your child's media use (TV, smartphone, personal computer, tablet, etc.)?<br>- co-use, e.g. searching the internet together with the child, playing computer games or watching movies/series together.<br>- active mediation, e.g. explaining movie content, explaining potential dangers of the internet (security, data protection, etc.), helping with problems/uncertainties that arise<br>- restrictive mediation (non-technical), e.g. restricting time of use, prohibiting certain content/activities/networks<br>- monitoring, e.g., controlling content of media use (movies, websites), checking information the child shares on social media<br>- technical restriction, e.g., automatically shutting off wifi, other timers that limit time of use	never<br>rarely<br>sometimes<br>often<br>always
Screen time child	
How long does your child spend per day in his/her free time (excluding homework) on a weekday or on a weekend day in total in front of/with screen media (TV, smartphone, PC, tablet, etc.)?<br>- duration - weekday<br>- duration - weekend day	never,<br>0.5 hours<br>1 hour<br>1.5 hours<br>[…]<br>12 hours<br>> 12 hours
Screen time mother	
How long do you spend per day in your free time (excluding work) on a weekday or on a weekend day in total in front of/with screen media (TV, smartphone, PC, tablet, etc.)?<br>- duration - weekday<br>- duration - weekend day	never,<br>0.5 hours<br>1 hour<br>1.5 hours<br>[…]<br>12 hours<br>> 12 hours

Device ownership child	
Which of the following devices does your child own (in her/his own room)?<br>- smartphone<br>- laptop, tablet or similar<br>- personal computer<br>- TV, smart TV or similar	yes<br>no
Extracurricular activities child	
What types of sports did your child participate in during the past six months?<br>- sports organized in a club	yes<br>no
Which of the following hobbies does your child engage in?<br>- choir, orchestra or other musical group<br>- theater or dance group<br>- playing an instrument<br>- other organized activity (except sports club)	yes<br>no
